# Identification and Validation of a Novel Three Hub Long Noncoding RNAs With m6A Modification Signature in Low-Grade Gliomas

**DOI:** 10.3389/fmolb.2022.801931

**Published:** 2022-02-14

**Authors:** Quang-Huy Nguyen, Tin Nguyen, Duc-Hau Le

**Affiliations:** ^1^ School of Computer Science and Engineering, Thuyloi University, Hanoi, Vietnam; ^2^ Department of Computer Science and Engineering, University of Nevada, Reno, NV, United States

**Keywords:** low-grade gliomas, long noncoding RNA, N6-methyladenosine, prognostic index, hub genes, signature

## Abstract

It has been evident that N6-methyladenosine (m6A)-modified long noncoding RNAs (m6A-lncRNAs) involves regulating tumorigenesis, invasion, and metastasis for various cancer types. In this study, we sought to pick computationally up a set of 13 hub m6A-lncRNAs in light of three state-of-the-art tools WGCNA, iWGCNA, and oCEM, and interrogated their prognostic values in brain low-grade gliomas (LGG). Of the 13 hub m6A-lncRNAs, we further detected three hub m6A-lncRNAs as independent prognostic risk factors, including *HOXB-AS1, ELOA-AS1,* and *FLG-AS1*. Then, the m6ALncSig model was built based on these three hub m6A-lncRNAs. Patients with LGG next were divided into two groups, high- and low-risk, based on the median m6ALncSig score. As predicted, the high-risk group was more significantly related to mortality. The prognostic signature of m6ALncSig was validated using internal and external cohorts. In summary, our work introduces a high-confidence prognostic prediction signature and paves the way for using m6A-lncRNAs in the signature as new targets for treatment of LGG.

## Introduction

Gliomas are an umbrella term that includes the most common primary brain tumors of the central nervous system (CNS). According to their histopathological features, they are graded on a scale of I (the least malignant) to IV (the most malignant) by the World Health Organization (WHO) (Louis et al., 2007). Accordingly, patients with brain low-grade gliomas (LGG) are assigned to the WHO grade II-III gliomas and said to have a rare cancer type of CNS. Indeed, LGG is only held accountable for about 15% of all brain and CNS tumors (Lu et al., 2020). Histologically, unlike higher grade gliomas, LGG develops locally and slowly into the normal brain tissue instead of going outside the brain. Although LGG in terms of their biological behaviour is benign and good prognosis, they are most likely to transform into high-grade gliomas regardless of the traditional treatment approaches of LGG applied, such as surgical treatment and postoperative radiotherapy (Duffau and Taillandier, 2015). Therefore, it is a pressing need to detect LGG-related biomarkers for early recognition and diagnosis, and for development of individualized treatment approaches.

The heterogeneity of tumors is said to be caused by the accumulation of epigenetic alteration (Yang et al., 2019). Aberrant expression of long non-coding RNAs (lncRNAs) in cancers has been demonstrated being significant epigenetic regulatory molecules and effective biomarkers as well for early diagnosis and therapy (Bach and Lee, 2018; Camacho et al., 2018). LncRNAs represent the RNA molecules whose size is greater than 200 nucleotides and which are inability to be translated into a protein. Although lncRNAs are among one of the poorest understood molecules, several previous studies have investigated various lncRNAs and realized their roles in a wide range of biological processes, such as eluding immune destruction and surveillance, capacitating replicative immortality, and activating invasion and metastasis (Gutschner and Diederichs, 2012; Bach and Lee, 2018). For example, lncRNA *CCDC26* contributes significantly to higher mortality of LGG patients (Enciso-Mora et al., 2013). Also in LGG, genetic variation of lncRNA *CDKN2B-AS1* exerts as a LGG-promoting factor and influences susceptibility (Shi et al., 2013). Moreover, previous studies have paid too many attentions to the classification of high-grade gliomas or glioblastomas. Therefore, biomarkers for prognostic differentiation of patients with LGG have still left ambiguous. The goal of this study is to discover the central role of lncRNAs with N6-methyladenosine (m6A) modification in LGG.

m6A refers to DNA methylation at the N6 position of adenosine and its modification has been demonstrated being the post-transcriptional regulatory markers in various types of RNAs, including lncRNAs. Functionally, lots of evidence have reported that m6A modification of RNA was implicated in affecting hub gene expressions, thereby resulting in a series of malignant biological behaviors, such as tumorigenesis, invasion, and metastasis (Jia et al., 2013; Roignant and Soller, 2017). Importantly, there also exists m6A modification in lncRNAs. For example, in prostate cancer, its bone metastasis is associated largely with the regulation of lncRNA *NEAT1-1* with m6A modification on the Pol II ser2 phosphorylation, causing the initiation of the complex CYCLINL1/CDK19/NEAT1-1 (Wen et al., 2020). In the development of colorectal cancer by stabilizing *IGF2BP2*, it is related to the regulation of lncRNA *LINRIS* on the expression of *MYC* to affect the process of glycolysis (Wang et al., 2019). Therefore, studying m6A modification will give us further biological insight into the interplay between lncRNAs versus cancers.

This study discovered m6A-modified lncRNAs (m6A-lncRNAs) in LGG. Specifically, we identified hub m6A-lncRNAs signature and prognostic values, potentially giving rise to novel targets for future investigations.

## Materials and Methods

### Acquisition and Preprocessing of LGG Data

The lncRNA-seq expression profile and corresponding clinical features of LGG patients, including age, histological diagnosis, grade, gender, race, seizure history, first presenting symptom, first presenting symptom longest duration, and radiation therapy, were accessed from The Cancer Genome Atlas (TCGA) (https://portal.gdc.cancer.gov/). Especially, we divided the patient age into two groups using a threshold of 35 years of age, as the age distribution of LGG patients reached a peak at this number (Supplementary Figure S1). External cohort CGGA mRNA-seq-693 (Wang et al., 2015; Liu et al., 2018) was gained from Chinese Glioma Genome Atlas (http://www.cgga.org.cn/download.jsp) to validate the prognostic value of screened m6A-lncRNAs.

A total of 478 eligible patients with LGG were kept after using hierarchical clustering to exclude four potential outliers (Supplementary Figure S2), consisting of TCGA-HT-A5RA, TCGA-QH-A6CX, TCGA-S9-A6WM, and TCGA-TM-A7C3, and excluding the patients TCGA-P5-A5ET, TCGA-P5-A5EU, and TCGA-TQ-A7RS whose overall survival (OS) ≤ 0. We used the R tool biomaRt (version 2.46.1) (Durinck et al., 2005; Durinck et al., 2009) for accessing Ensembl annotation. All missing genes, being not in the biomaRt database, were removed. The identified genes with m6A modification were retrieved from the RMVar database (http://rmvar.renlab.org/index.html) (Luo et al., 2021). Then, we randomly split the parental TCGA cohort into two sub-cohorts, training set and test set, with the ratio of 7:3. We did not see any statistically significant differences between them (Table 1). As a result, the training set comprised 308 LGG samples, which were used for analysis of m6A-lncRNA signatures and generation of a prognostic risk model. A test set of 170 LGG samples was used for independent validation of the performance of the prognostic risk model.

**TABLE 1:** Clinical features of LGG patients in each dataset, including the TCGA data, training set, and test set. Statistical test used to compare all the clinical features between the training and test cohorts was Pearson’s chi-squared test.

Covariates	Overall TCGA cohort (*n* = 478)	Training set (*n* = 308)	Test set (*n* = 170)	*p*-value
Histological diagnosis				0.138
Astrocytoma	176 (36.8%)	123 (39.9%)	53 (31.2%)	
Oligoastrocytoma	125 (26.2%)	79 (25.6%)	46 (27.1%)	
Oligodendroglioma	177 (37.0%)	106 (34.4%)	71 (41.8%)	
Grade				0.295
G2	232 (48.5%)	146 (47.4%)	86 (50.6%)	
G3	245 (51.3%)	162 (52.6%)	83 (48.8%)	
unknown	1 (0.2%)	0 (0.00%)	1 (0.59%)	
Gender				0.607
Female	216 (45.2%)	136 (44.2%)	80 (47.1%)	
Male	262 (54.8%)	172 (55.8%)	90 (52.9%)	
Race				0.817
American indian or Alaska native	1 (0.2%)	0 (0.00%)	1 (0.59%)	
Asian	6 (1.3%)	4 (1.30%)	2 (1.18%)	
Black or African American	21 (4.4%)	14 (4.55%)	7 (4.12%)	
White	441 (92.3%)	284 (92.2%)	157 (92.4%)	
unknown	9 (1.9%)	6 (1.95%)	3 (1.76%)	
Seizure history				0.848
No	160 (33.5%)	102 (33.1%)	58 (34.1%)	
Yes	285 (59.6%)	186 (60.4%)	99 (58.2%)	
unknown	33 (6.9%)	20 (6.49%)	13 (7.65%)	
First presenting symptom				0.227
Headaches	94 (19.7%)	67 (21.8%)	27 (15.9%)	
Mental Status Changes	36 (7.5%)	18 (5.84%)	18 (10.6%)	
Motor/Movement Changes	34 (7.1%)	24 (7.79%)	10 (5.88%)	
Seizures	234 (49.0%)	152 (49.4%)	82 (48.2%)	
Sensory Changes	17 (3.6%)	9 (2.92%)	8 (4.71%)	
Visual Changes	11 (2.2%)	7 (2.27%)	4 (2.35%)	
unknown	52 (10.9%)	31 (10.1%)	21 (12.4%)	
First presenting symptom longest duration				0.311
>181 Days	101 (21.1%)	74 (24.0%)	27 (15.9%)	
0–30 Days	203 (42.5%)	124 (40.3%)	79 (46.5%)	
31–90 Days	71 (14.9%)	46 (14.9%)	25 (14.7%)	
91–180 Days	35 (7.3%)	21 (6.82%)	14 (8.24%)	
unknown	68 (14.2%)	43 (14.0%)	25 (14.7%)	
Radiation therapy				0.062
No	104 (21.8%)	58 (18.8%)	46 (27.1%)	
Yes	126 (26.3%)	89 (28.9%)	37 (21.8%)	
unknown	248 (51.9%)	161 (52.3%)	87 (51.2%)	
Age				0.960
≤35	168 (35.1%)	109 (35.4%)	59 (34.7%)	
>35	310 (64.9%)	199 (64.6%)	111 (65.3%)	

### Identification of the Hub m6A-lncRNAs in LGG

In the training set, after intersecting lncRNAs with m6A-mediated genes, we picked computationally up a list of hub m6A-lncRNAs based on three state-of-the-art tools, including the weighted gene co-expression network analysis (WGCNA), an improved version of WGCNA (iWGCNA), and oCEM.

WGCNA (Langfelder and Horvath, 2008) first computed the Pearson’s correlation coefficient of the pairwise m6A-lncRNAs for constructing the similarity matrix. The similarity matrix was transformed into a weighted adjacency matrix after a soft threshold of β (Langfelder and Horvath, 2008) was identified. Next, the adjacency matrix was transformed into topological overlap matrix (TOM) measuring the connectivity between genes. Then, in terms of dissimilarity measure (1-TOM), average linkage hierarchical clustering was performed to cluster the genes with similar expression profiles for producing gene modules. The dissimilarity of module eigengenes (MEs) was calculated. We constructed the unsigned m6A-lncRNA co-expression network using the *blockwiseModules* function (v1.69). All tuning parameters were left as default. The minimum number of genes was set to 10 to ensure reliability of the results. Genes with a high intramodular connectivity were considered intramodular hub genes.

iWGCNA (Nguyen and Le, 2020) being an improved version of WGCNA was proposed previously by us. Its improvement was instead of hierarchically clustering the genes to detect co-expressed modules with default agglomeration method, we sought to determine the optimal method based on agglomerative coefficients. Consequently, Ward’s hierarchical clustering method was the best and the optimal soft-thresholding β was 7 (signed *R* ^2^ of 0.537, Supplementary Figure S3) in this study. All remaining parameters were kept as above.

oCEM (Nguyen and Le, 2022) was very recently developed by us to serve for the same task as the two tools above. The tool first detected principal components using either the independent component analysis (ICA) (Comon, 1994; Hyvärinen and Oja, 2000; Liebermeister, 2002) or the independent principal component analysis (IPCA) (Yao et al., 2012). Then, it turned the identified components into co-expressed modules by three optional post-processing steps attached with ICA and IPCA, including “ICA-FDR”, “IPCA-FDR”, and “ICA-Zscore”. For the two first options “ICA-FDR” and “IPCA-FDR”, we chose the probability threshold, called tail area-based false discovery rate (FDR), of 0.05, whereas the Z-score theshold of 0.5 was chosen for the last option “ICA-Zscore”. The m6A-lncRNAs at both extremes of the distribution of a certain co-expressed module over samples were considered hub ones.

### Identification of the Hub m6A-lncRNAs Prognostic Signature for LGG

A univariate Cox regression with a proportional hazards model (Andersen and Gill, 1982) was used to determine the correlation among expression levels of m6A-lncRNAs and patient outcome as follows:

$$\;\lambda \left(t\right)=\lambda_{o}\left(t\right)e^{x_{i}^{T}\beta }$$ (1)

where λ(t) is the hazard for patient *i* at the time *t*, λ(t) is a shared basedline hazard, *x* is an *n x p* matrix of covariate values (i.e., each row corresponds to a patient and each column a covariate), and *ß* is a fixed, length p vector. To do so, we first divided the expression levels of each hub m6A-lncRNAs into two groups, high expression and low expression, across LGG patients using a cut-off of median expression. Hub m6A-lncRNAs were then inputted into the R package “geneSA” (Nguyen and Le, 2020). The hub m6A-lncRNA was considered as an independent prognostic risk factor if adjusted *p*-value < 0.05 (Benjamini-Hochberg (Benjamini and Hochberg, 1995), two-sided). To avoid overfitting, we continued to recruit the Cox regression model with lasso penalty, which combats problems with *p >> n* by the use of an L1 (lasso) penalty in the Cox model (Tibshirani, 1997), to further select the best hub prognostic m6A-lncRNAs for construction of risk models. To do so, we split the training set (*n* = 308) by the ratio of 7:3 again, rendering the two parts of the parental training set including the training set (*n* = 198) and validation set (*n* = 110), respectively. 10-fold cross-validations were performed with the Harrell’s concordance index (C-index) as a measure to validate prediction performance. C-index ranges from 0.5 indicating absence of discrimination to 1.0 indicating perfect discrimination. Multivariate Cox regression analysis was then developed to determine the risk coefficients of prognostic markers for m6A-lncRNAs.

Hub m6A-lncRNAs signature (m6ALncSig) was constructed based on the multiple regression analysis of coefficients and expression levels of m6A-lncRNAs over the patients for prognosis prediction using the following equation:

$$\text{m}6\text{ALncSig risk score}=\;\overset{n}{\underset{i=1}{\sum }}coef\;m6ALncSig_{i}\times EXP\;m6ALncSig_{i}$$ (2)

where m6ALncSig risk score was the prognostic risk score of each LGG patients. $\text{coef m}6{\text{ALncSig}}_{\text{i}}$ represented the $i^{th}$ hub m6A-lncRNA multivariate Cox regression coefficient, whereas $\text{EXP m}6{\text{ALncSig}}_{\text{i}}$ represented the expression levels of corresponding hub m6A-lncRNA over the LGG patients.

### Identification and Verification of the Hub m6A-lncRNAs Survival Analysis for LGG

Patients were grouped into m6ALncSig low- and high-risk groups on the training set using the median risk score. Kaplan-Meier survival curve analysis was performed to compare differences in OS between the low- and high-groups. Hazard ratios (HR) and 95% confidence interval (CI) were used to analyze whether the prognoses of the two groups were significantly different. Cox log-rank and Gehan-Breslow-Wilcoxon *p*-values < 0.05 (two-sided) were used to validate statistical significance. A time-dependent subject receiver operating characteristic curve (ROC) analysis was utilized to compare the specificity and sensitivity of m6ALncSig risk score for prognosis of LGG patients. The above results were then validated using both test set and parental TCGA data.

### Construction of a Nomogram Based on the Hub Prognostic m6A-lncRNAs

Clinical variables showing significant associations with OS in multivariable analysis, combined with the m6ALncSig risk groups, were used to construct the one-, three-, and five-year nomograms for predictions of OS of patients. The nomograms were subjected to internal and external validations using the training and test cohorts, respectively. Bias-corrected internal validation was performed by evaluating discrimination and calibration under 250 bootstrap resamples. Discrimination, which is a measure of the probability of concordance between observed and predicted outcomes, was evaluated using C-index based on censored survival at one, three, and 5 years. The calibration curve was plotted to assess the relationship between the predicted probabilities and the actual probabilities of patient OS at one, three, and 5 years. The predictions should fall on a diagonal 45^o^ line of the plot in a perfectly calibrated model. The same methods were performed on the test cohort for external validation.

## Results

### Identification of 13 Hub m6A-lncRNAs for LGG

We received a list of 4,214 lncRNAs from the TCGA lncRNAs expression profile after clearing invalid lncRNAs (as described in the section “*Materials and Methods*” above) and a list of 850 m6A-lncRNAs from the RMVar database. Intersecting these lists, a total of 18 m6A-lncRNAs were kept for the hub lncRNAs identification process.

In the TCGA training cohort, 308 LGG patients alongside the 18 m6A-lncRNAs became the input of the three co-expressed module identification tools, including WGCNA, iWGCNA, and variations of oCEM. Table 2 shows a set of hub m6A-lncRNAs predicted by each method. Unfortunately, WGCNA did not identify any hub lncRNAs. In contrast, iWGCNA, oCEM with ICA-FDR, oCEM with ICA-Zscore and oCEM with IPCA-FDR indicated five, two, nine, and two hub m6A-lncRNAs, respectively. Taken together, we obtained a set of 13 hub m6A-lncRNAs in total.

**TABLE 2:** A set of 13 hub m6A-lncRNAs predicted by the three co-expressed module identification tools, including WGCNA, iWGCNA, and the three variations of oCEM.

	WGCNA	iWGCNA	oCEM with ICA-FDR	oCEM with ICA-Zscore	oCEM with IPCA-FDR
hub m6A-lncRNAs	-	ELOA-AS1	FLG-AS1	ZNF761	KRT73-AS1
	-	ZNF518A	HMGN3-AS1	HMGN3-AS1	ZNF761
	-	BCAR3-AS1	-	PABPC4-AS1	-
	-	ZNF571-AS1	-	OR51B5	-
	-	HMGN3-AS1	-	FLG-AS1	-
	-	-	-	BCAR3-AS1	-
	-	-	-	LINC00571	-
	-	-	-	KRT73-AS1	-
	-	-	-	HOXB-AS1	-

### Identification of the Three Hub m6A-lncRNAs Prognostic Signature for LGG

**FIGURE 1:**
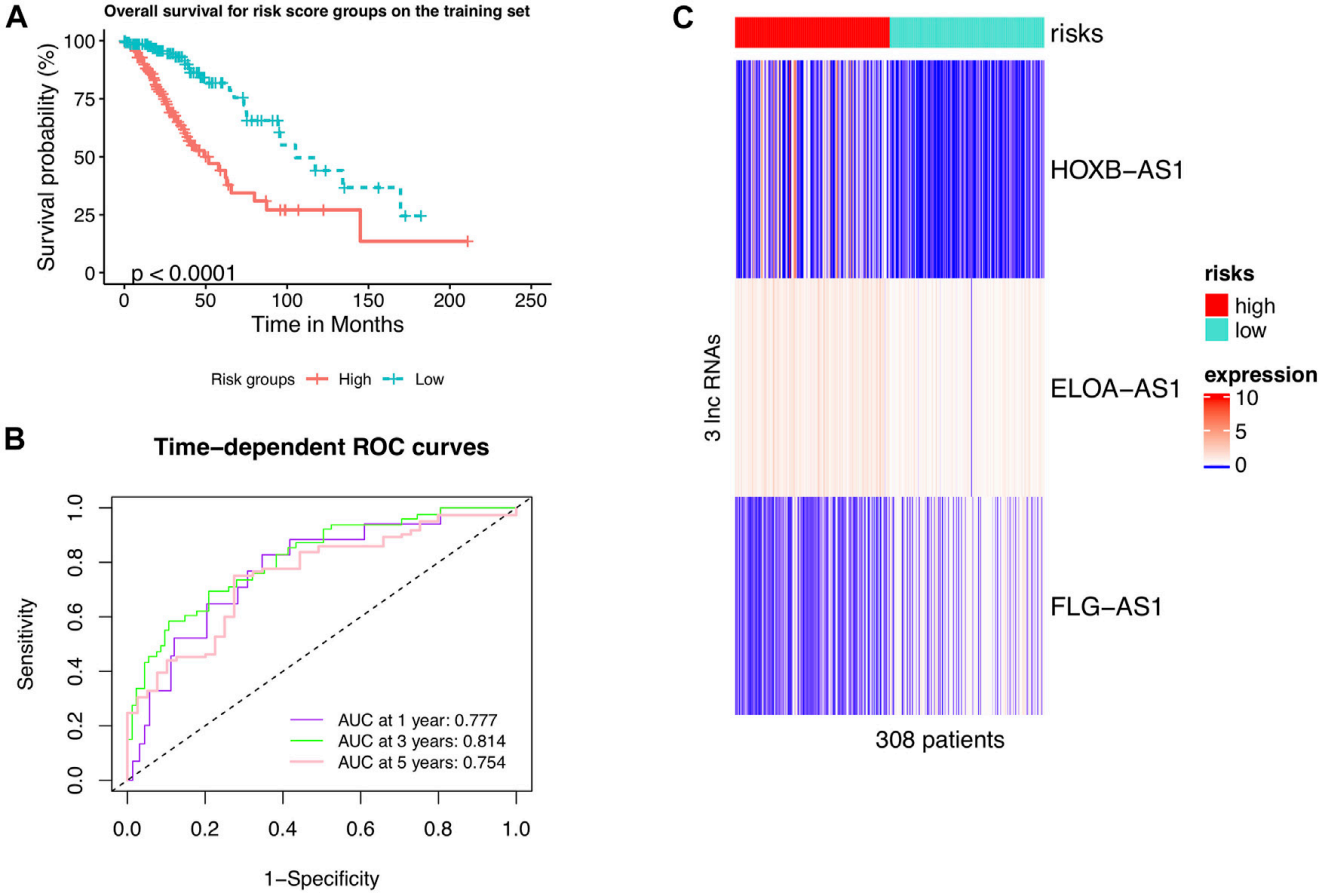
LncRNAs signature of the m6A modification used to predict outcomes in the training set. **(A)** Kaplan-Meier curve of m6ALncSig-predicted OS of low- or high-risk patients in the training set. **(B)** Time-dependent ROC curves of m6ALncSig at one, three, and 5 years. **(C)** Variations in expression levels of the three hub m6A-LncRNAs between the two risk groups.

A univariate Cox risk regression profiling was first performed on the 13 hub candidates to screen for independent prognostic risk factors, rendering three hub m6A-lncRNAs *HOXB-AS1, ELOA-AS1,* and *FLG-AS1*. Interestingly, the Cox regression model with lasso penalty using these three hub m6A-lncRNAs verified that they were related to patient outcome with the C-index of 0.739. Then, they were fed into a multivariate Cox risk regression model to construct the hub m6A-LncRNAs prognostic signature, m6ALncSig. Table 3 shows the outcome of the analyses. Accordingly, lower expression of *FLG-AS1* and higher expression of *ELOA-AS1* and *HOXB-AS1* were significantly associated with shorter survival. The m6ALncSig risk score was calculated based on the three independent prognosis-associated LncRNAs using the formula: m6ALncSig risk score = [0.604 x *EXP* HOXB-AS1] + [0.461 x *EXP* ELOA-AS1] + [−0.646 x *EXP* FLG-AS1]. The median m6ALncSig risk score was 0.276. This median value was used as the threshold to stratify LGG samples into low-risk group (*n* = 154) and high-risk group (*n* = 154). It was not surprised to see that tumors in the low-risk group had significantly better OS than tumors in the high-risk group (Figure 1A; Cox log-rank test: *p*-value < 0.01 and Gehan-Breslow-Wilcoxon test: *p* < 0.01; two-sided), implying that the prognostic lncRNAs signature used for risk score prediction was effective. Survival analyses also reported that the 1-year survival rate among high-risk LGG patients was approximately 0.898 with its 95% CI of 0.849–0.950, whereas the 1-year survival rate for low-risk LGG patients was approximately 0.986 with its 95% CI of 0.966–1.000. Besides, the 3-year survival rate for high-risk LGG patients was approximately 0.635 with its 95% CI of 0.545–0.740, while those numbers for patients with low-risk LGG was approximately 0.932 with its 95% CI of 0.883–0.984. Finally, the 5-year survival rate for high-risk LGG patients was approximately 0.442 with its 95% CI of 0.329–0.593, whereas the low-risk LGG patients had a 5-year survival rate of approximately 0.818 with its 95% CI of 0.727–0.922. Figure 1B illustrates time-dependent ROC curves of m6ALncSig over time for one, three, and 5 years reporting an AUC of 0.777, 0.814, and 0.754, respectively.

**TABLE 3:** Results of univariate and multivariate Cox regression model. Down regulation was the reference state. *p*-values were adjusted by the Benjamini-Hochberg procedure and called Q.values. Abbreviation: HR, Hazard ratio; 95% CI, 95% confidence intervals; Coef, multivariate Cox regression coefficient.

Gene	Univariate cox regression				Multivariate cox regression			
	HR	95% CI	*p*.value	Q.value	Coef	HR	95% CI	*p*.value
FLG-AS1	0.51	0.322–0.808	<0.01	0.042	−0.646	0.524	0.330–0.833	0.010
HOXB-AS1	1.92	1.215–3.035	<0.01	0.028	0.604	1.829	1.154–2.900	0.049
ELOA-AS1	1.734	1.100–2.733	0.016	0.049	0.461	1.585	1.002–2.507	<0.01

We further drew a heat map to show the differences in expression levels of the four hub m6A-LncRNAs between the two risk groups (Figure 1C). Interestingly, the two m6A-lncRNAs *FLG-AS1* and *HOXB-AS1* were steeply lowly expressed in the high- and low-risk group, respectively. Also, high-expression levels of the leftover lncRNA, *ELOA-AS1*, was more frequent in the high-risk group than in the low-risk group.

### Validation of the Three Hub lncRNAs Signature for Prognosis Using the Test Set and TCGA Data

**FIGURE 2:**
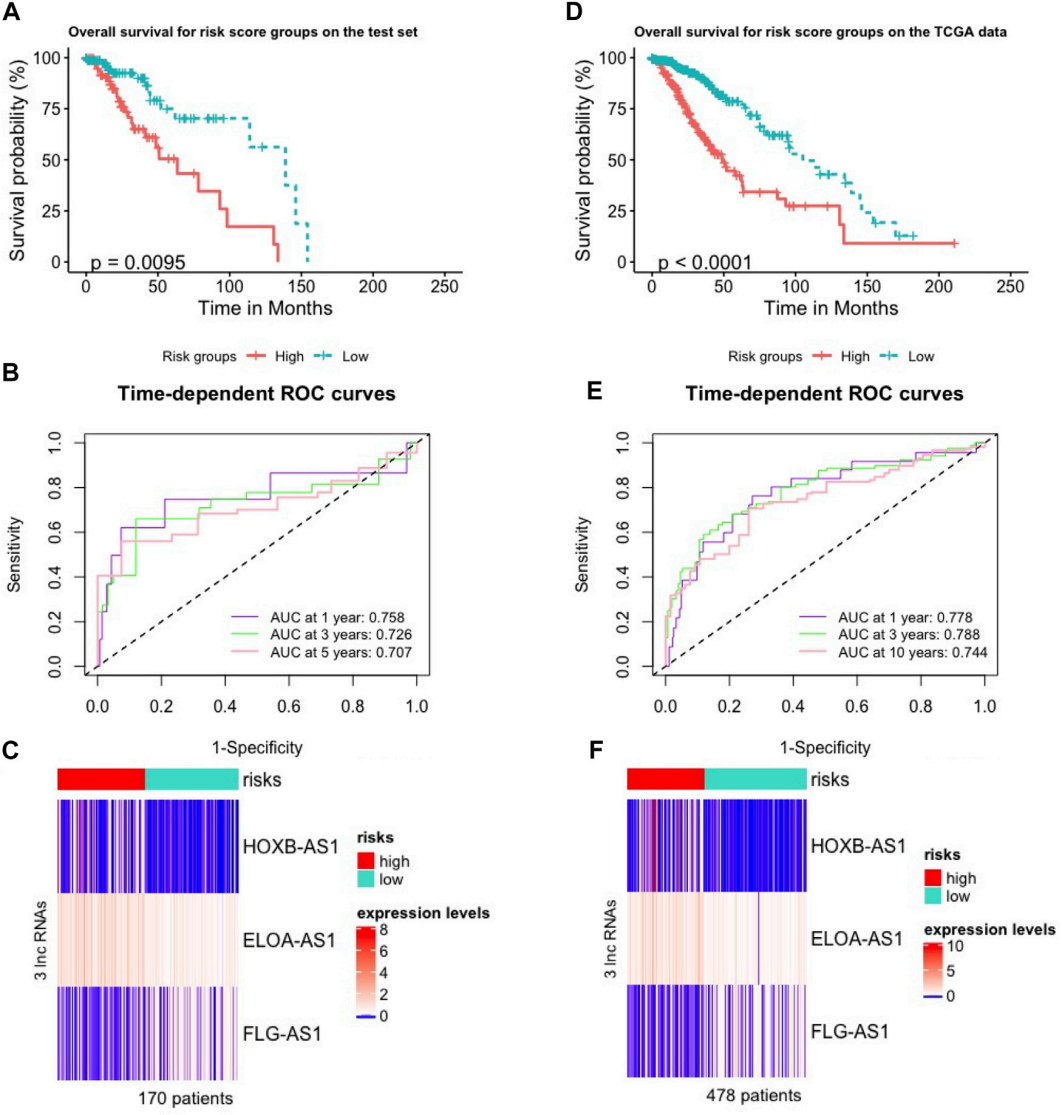
Validation of the m6A-lncRNA signature used to predict outcomes in the test and TCGA sets. **(A,D)** Validation of OS in low- or high-risk patients predicted by m6ALncSig with Kaplan-Meier estimates on the test set **(A)** and the parental TCGA data **(D)**. **(B,E)** Time-dependent ROC curves of GILncSig at one, three and 5 years on the test set **(B)** and the parental TCGA data **(E)**. **(C,F)** Variations in expression levels of the three hub m6A-LncRNAs between the two risk groups on the test set **(C)** and the parental TCGA data **(F)**.

We validated the m6ALncSig model on the test set including 170 LGG samples to test its prognostic performance. In order to do so, we took the same m6ALncSig and risk score threshold above to this case, rendering the low-risk group (*n* = 88) and high-risk group (*n* = 82). Kaplan-Meier curves showed that patients in the low-risk group displayed significantly higher OS compared with their counterparts in the high-risk group again (Figure 2A; Cox log-rank test: *p*-value < 0.01 and Gehan-Breslow-Wilcoxon test: *p* < 0.01; two-sided). Survival analyses showed that the one-, three-, and five-year survival rates of LGG patients in the high-risk group were approximately 0.919, 0.651, 0.505 with their 95% CIs of 0.859–0.983, 0.525–0.807, and 0.351–0.727, respectively. The one-, three-, and five-year survival rates of LGG patients in the low-risk LGG group were approximately 0.974, 0.899, and 0.750 with their 95% CIs of 0.940–1.000, 0.822–0.983, and 0.614–0.917, respectively. Besides, the time-dependent ROC curves of m6ALncSig over time for one, three, and 5 years on the test set showed AUC values of 0.758, 0.726, and 0.707, respectively (Figure 2B). Figure 2C illustrates the heatmap showing the same phenomena as the above results on the training set (Figure 1C).

We did the same things on the parental TCGA data (478 patients) as on the test set. As a result, we first received the low-risk group (*n* = 271) and high-risk group (*n* = 207). Then, OS analysis showed that showed that patients in the high-risk group was significantly poorer survival than their counterparts in the low-risk group (Figure 2D; Cox log-rank test: *p*-value < 0.01 and Gehan-Breslow-Wilcoxon test: *p* < 0.01; two-sided). The survival rates at one, three, and 5 years for patients with high-risk LGG were approximately 0.893, 0.624, 0.423 with their 95% CIs of 0.849–0.938, 0.545–0.716, and 0.322–0.555, respectively. The survival rates at one, three, and 5 years for patients with low-risk LGG were approximately 0.983, 0.902, and 0.788 with their 95% CIs of 0.967–1.000, 0.856–0.949, and 0.714–0.870, respectively. The one-, three-, and five-year time-dependent ROC curves of the TCGA data showed AUC values at 0.778, 0.788, and 0.744, respectively (Figure 2E). Frequency of expression levels of the four hub m6A-lncRNAs reported the same results in the heatmap (Figure 2F).

### Nomogram Development and Validation

**FIGURE 3:**
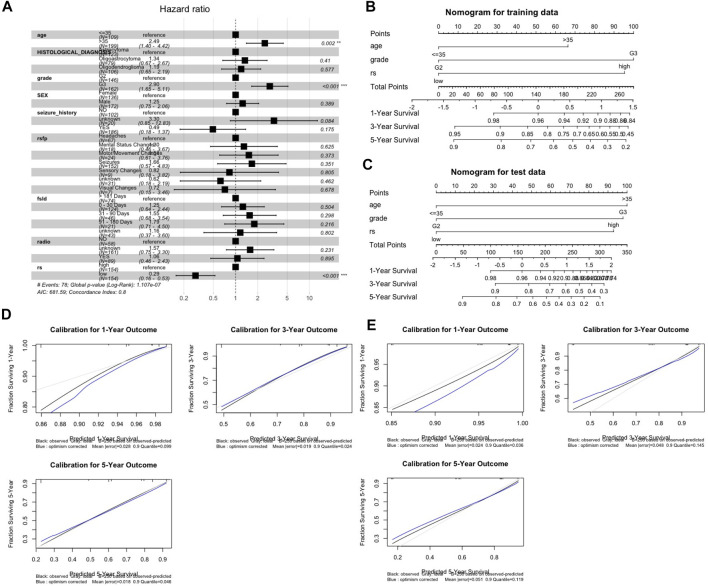
Nomogram development and validation. **(A)** Forest plot for identification of risk covariates. **(B,C)** One-, three-, five-year nomogram plots on the trainning set **(B)** and the test set **(C)**. **(D,E)** Evaluation of nomograms using calibration curves on the training set **(D)** and the test set **(E)**. Abbreviation: rsfp, First presenting symptom; fsld, First presenting symptom longest duration; radio, radiation therapy; rs, m6ALncSig risk group.

Three prognostic factors, including the age group, grade and m6ALncSig risk group, that witnessed significant associations with OS in the training cohort were included in the final predictive model (Figure 3A) for the construction of the one-, three-, and five-year nomogram plots. The discriminative ability of these nomogram plots on the training cohort (Figure 3B) was evaluated using C-index, which resulted in C-indices of 0.768, 0.766, and 0.765 with their 95% CIs of 0.711–0.820, 0.707–0.802, and 0.712–0.814, respectively. Similarly, for the test cohort, the nomogram plots (Figure 3C) rendered C-indices were 0.753, 0.751, and 0.753 with their 95% CIs of 0.659–0.832, 0.652–0.830, and 0.674–0.843, respectively. The calibration plots for internal and external validation displayed good agreement between the predicted one-, three-, and five-year OS probabilities and actual observations, with all the observed probabilities within 95% CI of the predicted probabilities (Figures 3D,E). The calibration plots illustrated that the nomograms were well calibrated.

### Verification of the Survival Signature for the Three Hub m6A-lncRNAs Using the CGGA Dataset

There were only two out of the three m6A-lncRNAs in m6ALncSig (*HOXB-AS1* and *FLG-AS1*) included in CGGA. Therefore, we only explored the relationship between these two lncRNAs and the OS of LGG patients using the CGGA dataset. No related external data were found to validate the prognostic value of *ELOA-AS1*. Consistently, lower expression of *FLG-AS1* and higher expression of *HOXB-AS1* were significantly correlated with patient outcome (Table 4) independent of patient cohort and data platforms.

**TABLE 4:** Validation of the survival signature for HOXB-AS1 and FLG-AS1 within the CGGA dataset. Down regulation was the reference state.

lncRNA genes	HR	95% CI	*pP*.value	Q.value
HOXB-AS1	1.569	1.285–1.916	<0.01	<0.01
FLG-AS1	0.687	0.563–0.838	<0.01	<0.01

## Discussions and Conclusion

Higher grade gliomas is one of the major cause of poor clinical outcome and higher mortality for patients with brain cancer in general. Irrespective of the traditional treatment approaches of LGG we apply, LGG patients are still most likely to transform into this dangerous stage (Duffau and Taillandier, 2015). Therefore, detection of LGG-related biomarkers is extremely necessary for early recognition and diagnosis, and for development of individualized treatment approaches. Moreover, aberrant expression of lncRNAs with m6A have been implicated in a series of malignant biological behaviors, such as tumorigenesis, invasion, and metastasis (Jia et al., 2013; Roignant and Soller, 2017; Bach and Lee, 2018; Camacho et al., 2018). However, only a few studies have explored the role of m6A-lncRNAs on prognosis of patients with LGG. Additionally, hub m6A-lncRNAs can be regarded as central factors for investigating the complex biological mechanism of cancer in general and LGG in particular.

In this study, by using the three advanced co-expression identification tools, including WGCNA, iWGCNA, and oCEM, we first computationally discover a total of 13 hub m6A-lncRNAs. Then the Cox regression analysis is performed to identify the three hub m6A-lncRNAs correlated closely with the prognosis of LGG patients, including *ELOA-AS1, HOXB-AS1,* and *FLG-AS1.* We continue to confirm this result by the Lasso-Cox model in order to prevent overfitting and gain the same result. To the best of our knowledge, with the exception of the lncRNA *FLG-AS1*, two out of the remainer lncRNAs (*ELOA-AS1* and *HOXB-AS1*) are identified newly, related to OS of LGG patients. Accordingly, lower expression of *FLG-AS1* and higher expression of *ELOA-AS1* and *HOXB-AS1* were significantly associated with shorter survival. The prognosis of *HOXB-AS1* and *FLG-AS1* are validated externally in the CGGA dataset. Next, m6ALncSig containing these three hub m6A-lncRNAs is constructed. Based on the m6ALncSig model, we cluster LGG patients into low- and high-risk groups, in which the high-risk patients experience significantly shortened life expectancy on the training set which was validated using the independent testing set. On the one hand, the risk-risk model was validated by Kaplan-Meier curve and ROC curve. One the other hand, one-, three- and five-year nomogram plots report that the model is a good predictor of prognosis for OS of LGG patients. The C-index and calibration curve further confirm that the model is accurate.

However, we acknowledge that this study still manifests several restrictions. Firstly, despite inclusion of internal and external validations, it is required to have more independent datasets to validate m6ALncSig ensuring its reproducibility and robustness. Especially, the prognostic values of ELOA-AS1 cannot be valdiated due to lack of the external cohorts. Secondly, deeper understandings of roles of the three identified m6A-lncRNAs will benefit a lot from using flow cytometry, PCR, or IHC. Thirdly, we do not conduct further animal studies and cellular experiments to test the predictive accuracy of our signature as well as to discover the molecular mechanisms of m6A-lncRNAs.

In summary, through this study, many exciting results have been identified, from identifying 13 hub m6A-lncRNAs in LGG based on WGCNA, iWGCNA, and oCEM, then constructing and validating the m6ALncSig model according to the three hub m6A-lncRNAs linked with prognosis, to exploring their biological signatures and prognostic values. Hopefully, the findings of the present work can help improve the power of existing diagnosis and prognosis prediction for LGG patients.

## Data Availability

The datasets presented in this study can be found in online repositories. The names of the repository/repositories and accession number(s) can be found in the article/Supplementary Material.
