# Supplementary material for: Identification and Validation of a Novel Three Hub Long Noncoding RNAs With m6A Modification Signature in Low-Grade Gliomas
Source: Front Mol Biosci. 2022 Feb 14;9:801931. doi: 10.3389/fmolb.2022.801931 (PMC8882983; doi:10.3389/fmolb.2022.801931)
Supplement: Supplementary file 1 [file DataSheet1.docx]

**Supplementary Figure**

**Supplementary Figure S1. Age distribution of LGG patients.** The highest age density of patients stood at 35.

**Supplementary Figure S2. Detection and deletion of outliers.** Hierarchical clustering was used to detect outliers.

**Supplementary Figure S3. Optimal soft-thresholding ß.** The optimal number of soft-thresholding ß was 7.
